# The Effects of Color Therapy on Fatigue, Depression, and Quality of Life in Parkinson's Patients: A Randomized Controlled Trial

**DOI:** 10.1002/brb3.71348

**Published:** 2026-03-26

**Authors:** Kübra Coskun, Gulcan Bahcecioglu Turan

**Affiliations:** ^1^ Department of Internal Medicine Nursing, Institute of Health Sciences Fırat University Elazig Turkey; ^2^ Department of Nursing Faculty of Health Sciences Fırat University Elazığ Turkey

**Keywords:** Color therapy, depression, fatigue, nurse, Parkinson's, Quality of Life

## Abstract

**Background:**

When compared with healthy people, fatigue, depression, and a lower quality of life are frequently experienced by Parkinson's patients. Color therapy has effects such as reducing depression and improving focusing problems.

**Aim:**

Examining the effects of color therapy on fatigue, depression, and quality of life in Parkinson's patients constitutes the aim of the present study.

**Method:**

This randomized controlled trial was conducted with 60 Parkinson's patients (30 intervention, 30 control). In the first and second weeks, the intervention group received color therapy 3 days per week, consisting of 15‐min exposures to purple, yellow, and orange light (totaling 45 min per session). No intervention was performed during the third and fourth weeks. In the fifth and seventh weeks, a single weekly session was administered, applying each color for 15 min (totaling 45 min), while no intervention was carried out in the sixth week. Post‐test data were collected in the eighth week. The control group received no intervention. Data collection tools included a “Patient Information Form,” “Parkinson Fatigue Scale (PFS‐16),” “Beck Depression Inventory (BDI),” and “SF‐36 Quality of Life Scale.”

**Results:**

The total mean score and sub‐dimension scores of PFS‐16 and the total mean score of BDI decreased statistically significantly in the intervention group (p = 0.05). The intervention also caused a significant increase in SF‐36 quality of life total score and sub‐dimension mean scores of the intervention group (p < 0.05).

**Conclusion:**

Color therapy application was found to decrease the level of fatigue and depression and improve the quality of life in Parkinson's patients.

## Graphical abstract

**Study Design**: A randomized controlled trial conducted with 60 Parkinson's patients (30 intervention, 30 control).

**Intervention**: An 8‐session color therapy program using purple, yellow, and orange lights (15 minutes each; total 45 minutes per session).

**Protocol**: 3 sessions/week in the first two weeks, followed by 1 session/week in the fifth and seventh weeks, with specific washout periods to monitor residual effects.

**Outcomes**: Significant reduction in fatigue (PFS‐16) and depression (BDI) levels compared to the control group (p<0.05).

**Quality of Life**: Statistically significant improvement in all sub‐dimensions of the SF‐36 Quality of Life Scale (p<0.05).

**Conclusion**: Color therapy is an effective, non‐invasive supportive intervention for managing non‐motor symptoms and enhancing the quality of life in Parkinson's disease.

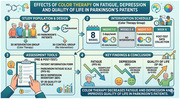

## 1 Introduction

**Affecting approximately 2–3% of individuals over the age of 65, Parkinson's disease (PD) is the second most common neurodegenerative disease after Alzheimer's disease** (Hayes 2019; Poewe et al. 2017). Globally, the number of individuals with PD increased significantly between 1990 and 2015, largely attributed to the aging population (Hayes 2019). It is estimated that there will be 12.9 million Parkinson's patients by 2040 (Dorsey et al. 2018). In Turkey, the prevalence is reported to be 1–3% for individuals older than 65 and 3–5% for those older than 85 (Emek‐Savaş et al. 2017).

Although previously believed to be a disease affecting the motor system, Parkinson's disease is now considered a multidimensional disease with autonomic, behavioral, cognitive, and sensory involvement in addition to motor symptoms. Diagnosis is difficult in the early stages of the disease, which can present with symptoms so varied and complex that treatment may be delayed (Çakmur 2011). Parkinson's disease usually has an insidious onset and progresses over time, increasing the severity of symptoms. During this period, complaints such as fatigue, weakness, muscle weakness, and difficulty in walking are common in patients (Çakmur 2011). In this process, accompanied by both specific and nonspecific symptoms, anxiety, depression, and fatigue levels increase, and quality of life decreases significantly (Çakmur 2011). Activities of daily living are difficult to perform for patients. In raising public awareness and increasing awareness about the disease, patient education and nursing care are vitally important. Assessing the quality of life has a critical importance in determining the care needs of individuals (Civil 2018).

Health‐related attitudes and behaviors of Parkinson's patients are significantly influenced by seeking health, traditional health beliefs, lifestyle, cognitive and emotional processes, cultural factors, and previous experiences related to the disease (Talhaoğlu 2021). Color therapy (chromotherapy), which is one of the non‐pharmacological treatment approaches for this disease, is not widely used in clinical practice despite the advanced diagnostic and treatment possibilities of modern medicine (Büker and Elinç 2023). However, with the increasing interest in non‐pharmacological complementary therapies, awareness of color therapy is also increasing. Colors influence the psychological and physiological states of individuals directly and play a role in preventing some diseases and alleviating their symptoms by creating balancing effects on the nervous system (Bal 2019). There is research in literature indicating that color therapy provides positive effects such as reducing levels of stress, eliminating distraction, increasing alertness, and reducing behavioral problems such as aggression (Nichol 2001). In fact, chromotherapy practices in institutions such as hospitals, schools, and prisons have been shown to have positive effects on environmental conditions. For example, studies using certain shades of red in the treatment of migraines and pink in the reduction of stress have yielded successful results (Bal 2019).

Although color therapy is not yet widely accepted in modern medicine, the effects of colors on psychological well‐being can be considered a supportive method in nursing care. Researchers working in this field have stated that color balance is very important in the well‐being of individuals both physically and psychologically, and physical symptoms that occur when this balance is disturbed can be reduced by rebalancing with appropriate colors (Büker and Elinç 2023). There are few studies on color therapy in literature that have been conducted on different patient groups (Bal 2019; Guseva et al. 2021; Kniazeva et al. 2006) although there are no studies addressing color therapy practices for Parkinson's patients. In this regard, the present study aims to examine the effects of color therapy on fatigue, depression, and quality of life in Parkinson's patients and thus to fill a gap in the literature.

Research Hypotheses

**H0**: Color therapy has no significant effects on fatigue, depression, and quality of life in Parkinson's patients.
**H1**: Color therapy has a significant effects on fatigue, depression, and quality of life in Parkinson's patients.

## 2 Methods

### 2.1 Type of Research

The study was conducted as a randomized experimental research with a pretest‐posttest control group.

### 2.2 Population and Sample of Research

Parkinson's disease patients admitted to the Neurology Outpatient Clinic and Clinic of Fırat University Hospital between November 2023 and June 2024 constituted the population. The sample included randomly selected patients who met the research criteria (Inclusion criteria; having a Parkinson's disease diagnosis for at least 6 months, having a scale score >3.3 on the Parkinson's Fatigue Scale, and having a total score between 24 and 30 on the Mini Mental State Test). Exclusion criteria: Parkinson's patients who had taken sleeping pills or sedative medication, those who had undergone brain pacemaker surgery, those who had physical illness or cognitive impairment at a level that prevented understanding the training, and those who had a diagnosis of psychiatric illness, who agreed to participate in the study. Sample size was determined with a priori power analysis using the G‐Power 3.1.9.4 program. The minimum number of patients to be included in the study was 56 when the effect size was 0.8 according to the t test in independent groups in Cohen's effect size table (Cohen et al. 2013); the confidence interval was 95% (Faul et al. 2007), the significance level was 0.05 (Faul et al. 2007), and the power was 0.90 (Çapık 2014). Based on this, it was determined that there should be at least 28 patients in each group. While 75 patients were reached, 60 Parkinson's patients were included in the sample since six did not meet the research criteria and nine did not agree to participate.

#### 2.2.1 Randomization

**FIGURE 1:**
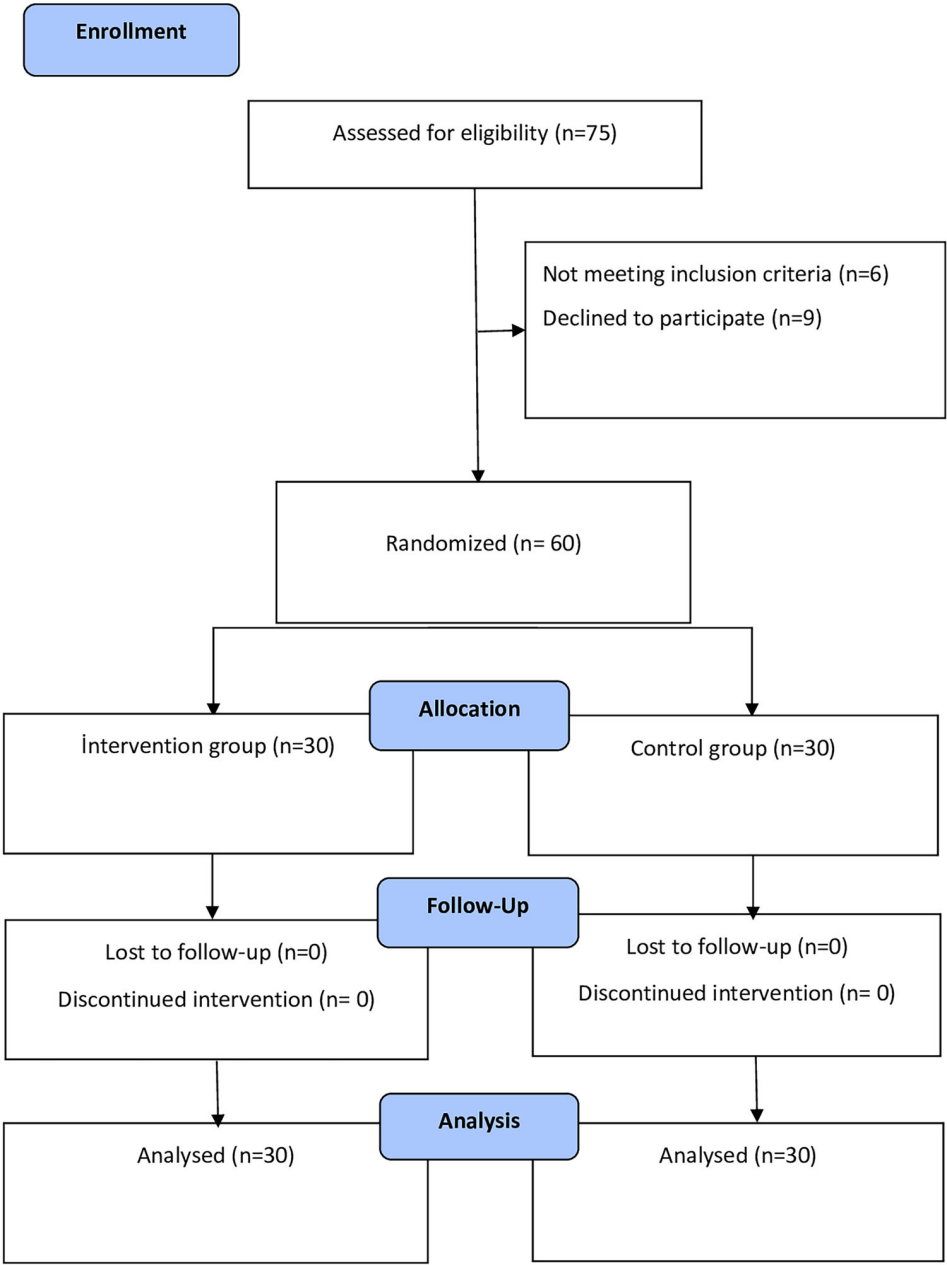
Flow  Diagram of Study.

In the study, the Random Integer Generator method in the Numbers sub‐heading of the random.org website (https://www.random.org/#numbers) was used to create a single group column between 1 and 60 in the system. The numbers 1 and 2 were used in the column. At the beginning of the study, group numbers were determined by an independent researcher through drawing lots. The odd number (1) was determined to be the intervention, while the even number (2) became the control group. Blinding was not possible because of the nature of the study. Only statistical blinding was carried out. When the research was completed, the data were analyzed by a statistician independent of the research who did not know groups 1 and 2, and the findings were reported (See Figure 1 Flow Diagram of the Study). CONSORT guidelines were followed.

### 2.3 Data Collection Tools

Patient Information Form, Parkinson's Fatigue Scale (PMS‐16), Beck Depression Scale (BDS), and SF‐36 Quality of Life Scale.

#### 2.3.1 Patient Information Form

This form includes questions such as age, gender, marital status, level of education, income, employment status, duration of diagnosis, hospitalization in the last year, use of psychological medication, stage of the disease, and family history of Parkinson's disease.

#### 2.3.2 Parkinson Fatigue Scale

Brown et al. developed the scale in 2005 for the assessment of fatigue in Parkinson's disease (2005). Çılga et al. (2017) conducted a Turkish validity and reliability study. The scale consists of 16 items with 5 responses (1 = strongly disagree, 5 = strongly agree). Total value is calculated by the average of the responses to all items (value range 1–5). A mean score above ‘3.3’ is considered fatigue. Cronbach's alpha internal coefficient of the PFS‐16 Turkish adaptation was found to be 0.94 (Çilga et al., 2017), whereas it was found to be 0.90 in the present study.

#### 2.3.3 Beck Depression Inventory

It was originally developed to determine the level of depression by Beck et al. (1961). Hisli conducted a Turkish validity and reliability study in 1989. (Hisli 1989). BDI is a Likert‐type scale with 21 questions, each scored between 0 and 3. Scores between 0 and 9 indicate minimal, 101 and 6 indicate mild, 17 and 29 indicate moderate, and 30 and 63 indicate severe depressive symptoms. A score of 17 or above indicates depression at a level that may require treatment. The Original scale's Cronbach's alpha internal consistency coefficient was 0.80 (Hisli 1989), while it was 0.87 in the present study.

#### 2.3.4 SF‐36 Quality of Life Scale

Ware et al. (1993) developed the SF‐36 Quality of Life Scale to evaluate health policies (Ware et al. 1993). It has 36 items and 8 subscales: physical functioning, physical role difficulties, emotional role difficulties, vitality, mental health, social functioning, pain, and general health perception. Koçyiğit et al. conducted a Turkish validity and reliability study in 1999. Item scores for each quality of life domain are coded and placed on a scale of 0–100 (0 being the worst, 100 being the best) by using algorithms. A Turkish validity and reliability study was conducted by Koçyiğit et al. (1999), and Cronbach's alpha values of the sub‐scales were found between 0.73 and 0.76 (Koçyiğit et al. 1999). In the present study, Cronbach's alpha values were found between 0.76 and 0.87.

### 2.4 Data Collection

First, an empty room in the outpatient clinic was provided by the researcher to interview patients. Patients who agreed to participate and who met the research criteria were informed about the study, and their verbal and written consents were taken. Before the intervention, patients in both groups were administered the “Patient Information Form,” “Parkinson's Fatigue Scale (PFS‐16),” “Beck Depression Inventory (BDI),” and “SF‐36 Quality of Life Scale” face‐to‐face by the researcher in approximately 15–20 min. At the end of the eighth week, both groups were re‐administered the PSI‐16, BDI, and SF‐36 Quality of Life Scale, and post‐test data were collected.

### 2.5 Intervention

#### 2.5.1 Intervention Group

The researcher received chromotherapy practitioner training conducted by the Continuing Practice and Research Centre before starting the practice. Color therapy was performed in a quiet, calm, convenient outpatient clinic and in an empty room allocated specifically for the intervention to minimize environmental distractions and ensure standardized application conditions.

**FIGURE 2:**
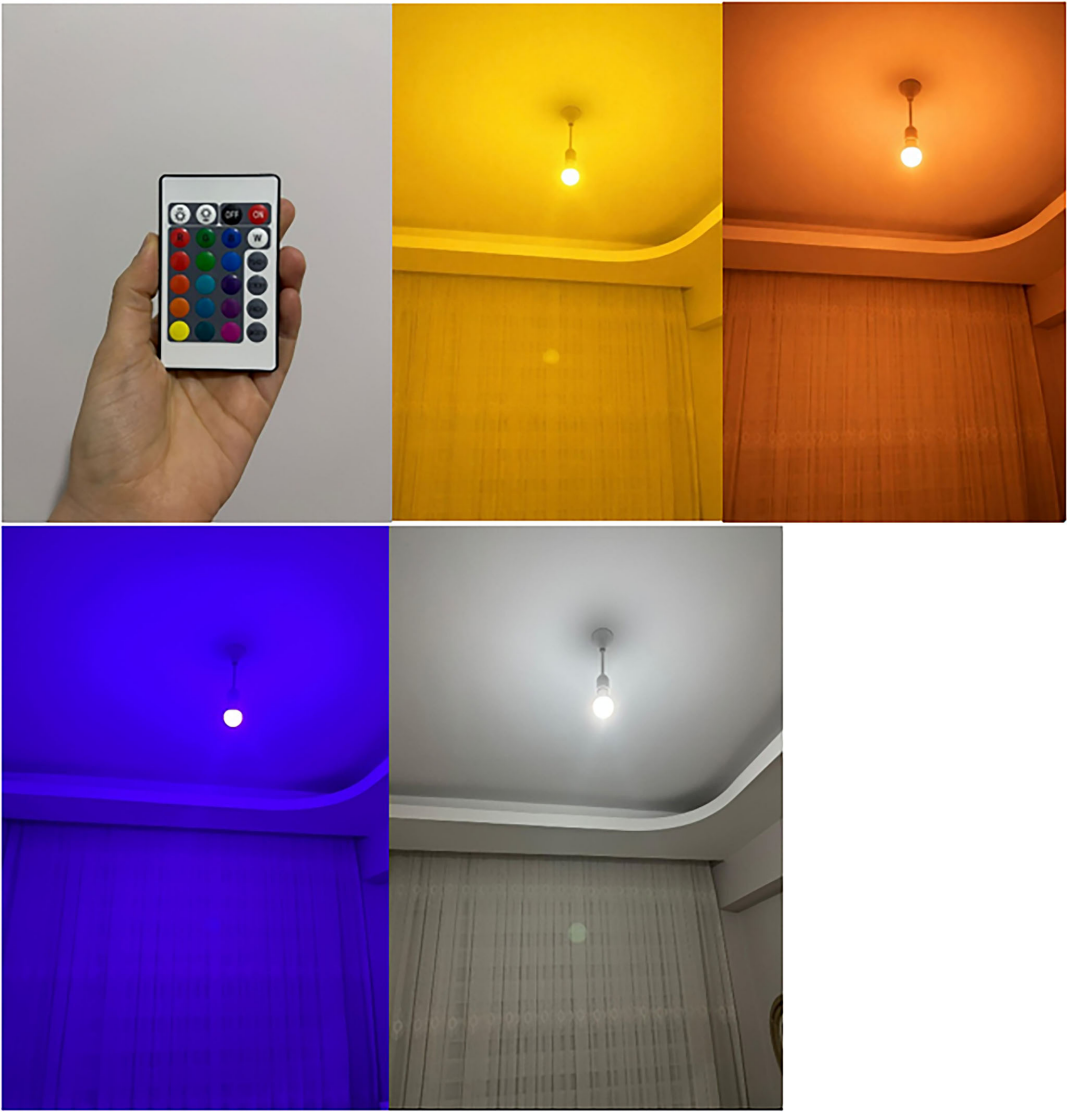
Multicolor LED lighting system and remote‐controlled device used for color therapy intervention showing selectable therapeutic colors (white, yellow, orange, and purple).

Color therapy was administered using a multicolor LED lighting system controlled via a remote‐operated device providing 13 selectable color options. The lighting system allowed standardized color exposure throughout all intervention sessions. The light source was positioned at a fixed and equal distance from each participant, and ambient room lighting was minimized to prevent interference with color perception. Device functionality and color output consistency were checked prior to each session to maintain intervention standardization and reproducibility. The multicolor lighting device and remote‐control system used in the intervention are presented in Figure 2.

Survey forms were applied to intervention group patients before color therapy and baseline measurements were obtained. During the implementation process, patients were informed about the purpose of color therapy, its potential benefits, and the procedures to be followed during therapy sessions (Andrews 2012).

In the first and second weeks, Parkinson's patients were exposed to purple, yellow, and orange lights 3 days per week. Each session lasted a total of 45 min, with 15 min dedicated to each color via the color‐lighting system. No intervention was applied during weeks three and four to observe the washout effect and persistence of the therapy. In the fifth and seventh weeks, the same color sequence (purple, orange, and yellow) was applied for 15 min each in a single weekly session (totaling 45 min). No intervention was performed in the sixth week. Intervention was intentionally withheld during weeks three, four, six, and eight to monitor residual therapeutic effects and to mitigate potential adverse psychological reactions, such as irritability or anger associated with overexposure (Andrews 2012). Post‐intervention measurements were obtained at the end of the eighth week, following a total of eight 45‐minute color therapy sessions.

#### 2.5.2 Control Group

Control group Parkinson's patients did not receive any intervention during the process. After the necessary information was given to the patients by the researcher, the survey forms were applied to 30 patients, and the first measurement was obtained. At the end of week 8, the survey forms were applied to the patients again without any intervention, and the second measurement was obtained.

### 2.6 Evaluation of Data

Data were analyzed using SPSS 27.0 software. Normality was assessed using skewness and kurtosis values (±2) and visual inspection of histogram graphs. Internal consistency of the scales was evaluated using Cronbach's alpha, and the results indicated that the scales were suitable for analysis. Descriptive characteristics of both groups were presented with frequency and percentage. Pearson chi‐square analysis was conducted to examine sociodemographic differences between the groups. Scale scores were summarized with mean and standard deviation values. To compare post‐intervention scores between groups, analysis of covariance (ANCOVA) was applied, with baseline (pre‐intervention) scores included as covariates to control for initial differences. All analyses were evaluated at a significance level of p < 0.05.

### 2.7 Ethical Considerations

The Declaration of Helsinki ethical guidelines were followed. Verbal and written permissions were received from the Interventional Research Ethics Committee of Fırat University, the institution where the study was conducted, and patients. Each patient was briefly informed about the reasons for conducting the study before starting the study, and written informed consent was obtained with an informed consent form. In addition, during the planning phase for the study, a number was obtained from ClinicalTrials, an official institution that reviews and evaluates experimental research at the international level and gives approval (ClinicalTrials.gov ID: NCT06154772).

## 3 Results

Pearson chi‐square analysis results indicated both groups were homogeneous in terms of gender, marital status, educational status, employment status, income, disease stage, psychological medication use, hospitalization in the last year, and family history of Parkinson's disease (p > 0.05). In the independent groups *t*‐test, no significant difference was found between the ages of patients in both groups; therefore, both groups were homogeneous in terms of age, duration of disease diagnosis, and mini mental status scores (p > 0.05) (Table 1).

**TABLE 1:** Distribution and comparison of the findings regarding the descriptive characteristics of the patients in the intervention and control groups (*n* = 60).

Variables		Groups				
		Intervention		Control		Test value and significance
		*N*	%	*N*	(%)	
Gender	Female	14	46.7	14	46.7	*χ* ^2^ = 0.00 p = 1.00
	Male	16	53.3	16	53.3	
Marital status	Married	28	93.3	30	1.00	*χ* ^2^ = 2.069 p = 0.150
	Single	2	6.7	—	—	
Educational status	Illiterate	—	—	1	3.3	*χ* ^2^ = 1.476 p = 0.871
	Literate	4	13.3	3	10.0	
	Primary	9	30.0	11	36.7	
	High school	16	53.3	14	46.7	
	Undergraduate and higher	1	3.3	1	3.3	
Employment status	Employed	10	33.3	10	33.3	*χ* ^2^ = 0.00 p = 1.00
	Unemployed	20	66.7	20	66.7	
Income level	Income<expense	1	3.3	—	—	*χ* ^2^ = 4.098 p = 0.129
	Income = expense	27	90.0	23	76.7	
	Income>expense	2	6.7	7	23.3	
Disease stage	Beginning	11	36.7%	10	33.3%	*χ* ^2^ = 0.088 p = 0.957
	Mid	12	40.0%	13	43.3%	
	Final	7	23.3%	7	23.3%	
Using psychological drug	Yes	14	46.7%	13	43.3%	*χ* ^2^ = 2.455 p = 0.117
	No	16	53.3%	17	56.7%	
Hospitalization in the past year	Yes	6	20.0%	13	43.3%	*χ* ^2^ = 3.774 p = 0.052
	No	24	80.0%	17	56.7%	
Familial history of Parkinson's	Yes	13	43.3%	8	26.7%	*χ* ^2^ = 1.832 p = 0.176
	No	17	56.7%	22	73.3%	
**Continuous variables**	**Mean± SD**		Min‐max	**Mean ± SD**	Min‐Max	
Age	59.70 ± 10.40		38‐76	63.10±8.59	44–81	*t* = 1.381 p = 0.173
Duration of Diagnosis	4.50 ± 3.57		3–15	3.30 ± 2.22	1–10	*t* = −1.565 p = 0.123
Mini Mental State Test	27.93 ± 1.48		25–30	27.37 ± 1.65	25–30	*t* = −1.399 p = 0.167

**p˂0.05** considered as statistically significant; χ^2^: Pearson Chi‐square test; t: Independent groups *t*‐test; SD: Standard Deviation.

ANCOVA was conducted to compare post‐intervention Parkinson Fatigue Scale scores between the intervention and control groups, controlling for baseline scores as a covariate. Baseline fatigue scores significantly influenced post‐intervention outcomes (F (1, 57) = 35.80, p < 0.001, *η* ^2^ = 0.386). After adjusting for the covariate, the intervention group showed significantly lower fatigue scores than the control group (F (1, 57) = 77.48, p < 0.001, *η* ^2^ = 0.576), indicating the effectiveness of the intervention (Table 2).

**TABLE 2:** ANCOVA Results for Post‐intervention Parkinson Fatigue Scale Scores adjusted for baseline values.

Variables	Mean ± SD	F	p	Partial *η* ^2^
**Group**		77.48	< 0.001	0.576
Control	3.78 ± 0.32			
Intervention	3.14 ± 0.32			
**Baseline score** **(Pre‐intervention)**		35.80	< 0.001	0.386

*Note*: Post‐intervention Parkinson Fatigue Scale scores were compared between groups using ANCOVA with baseline scores as covariates.

ANCOVA was conducted to compare post‐intervention Beck Depression Inventory scores between the intervention and control groups, controlling for baseline scores as a covariate. Baseline depression scores significantly influenced post‐intervention outcomes (F (1, 57) = 40.36, p < 0.001, *η* ^2^ = 0.415). After adjusting for the covariate, the intervention group showed significantly lower depression scores than the control group (F (1, 57) = 84.70, p < 0.001, *η* ^2^ = 0.598), indicating the effectiveness of the intervention (Table 3).

**TABLE 3:** Adjusted Post‐intervention Beck Depression Inventory Scores by group based on ANCOVA.

Variables	Mean ± SD	F	p	Partial *η* ^2^
**Group**		84.70	< 0.001	0.598
Control	36.30 ± 4.48			
Intervention	28.77 ± 3.32			
**Baseline score** **(Pre‐intervention)**		40.36	< 0.001	0.415

*Note*: Post‐intervention Beck Depression Inventory scores were compared between groups using ANCOVA with baseline scores as covariates.

Post‐intervention analysis of the SF‐36 Quality of Life subscales, adjusted for baseline scores using ANCOVA, revealed significant improvements in several domains among the intervention group compared to the control group. Specifically, participants in the intervention group demonstrated significantly higher scores in role emotional (p = 0.046), vitality (p < 0.001), mental health (p < 0.001), social functioning (p = 0.008), pain (p = 0.004), and general health (p = 0.012). In contrast, physical functioning (p = 0.170) and role physical (p = 0.839) did not show statistically significant differences between groups. Baseline scores contributed significantly to the variance in some domains, particularly physical functioning, role‐physical, and social functioning. These findings suggest that the intervention was effective in enhancing the psychological, social, and general health aspects of quality of life, whereas its impact on physical health‐related domains was limited (Table 4).

**TABLE 4:** Adjusted Post‐intervention SF‐36 Quality of Life Scale Subscale Scores by group based on ANCOVA.

Domain/Group	Mean ± SD	F	p	Partial *η* ^2^
Physical functioning				
Group		1.928	0.170	0.033
Control	25.67 ± 11.94			
Intervention	29.00 ± 20.27			
Baseline score (Pre‐intervention)		105.119	< 0.001	0.648
**Role physical**				
Group		0.042	0.839	0.001
Control	45.83 ± 26.33			
Intervention	42.50 ± 27.19			
Baseline score (Pre‐intervention)		16.316	< 0.001	0.223
**Role emotional**				
Group		4.143	0.046	0.068
Control	37.78 ± 25.87			
Intervention	52.22 ± 27.24			
Baseline score (Pre‐intervention)		1.547	0.219	0.026
**Vitality**				
Group		14.062	< 0.001	0.198
Control	45.33 ± 5.71			
Intervention	52.50 ± 9.98			
Baseline score (Pre‐intervention)		8.389	0.005	0.128
**Mental health**				
Group		16.436	< 0.001	0.224
Control	49.07 ± 8.91			
Intervention	58.53 ± 10.69			
Baseline score (Pre‐intervention)		7.665	0.008	0.119
**Social functioning**				
Group		7.676	0.008	0.119
Control	57.08 ± 13.80			
Intervention	67.50 ± 19.86			
Baseline score (Pre‐intervention)		33.248	< 0.001	0.368
**Pain**				
Group		9.257	0.004	0.140
Control	74.83 ± 12.85			
Intervention	84.92 ± 15.01			
Baseline score (Pre‐intervention)		6.089	0.017	0.097
**General health**				
Group				
Control	39.33 ± 4.50	6.659	0.012	0.105
Intervention	43.00 ± 6.38			
Baseline score (Pre‐intervention)		0.469	0.496	0.008

*Note*: Post‐intervention SF‐36 Quality of Life Scale Subscale scores were compared between groups using ANCOVA with baseline scores as covariates.

## 4 Discussion

Non‐pharmacological approaches stand out as an important complementary component in addition to pharmacological treatments for Parkinson's disease (Olanow et al. 2009). Although color therapy, one of the non‐pharmacological methods, is used in various health fields (Bal 2019; Guseva et al. 2021; Kniazeva et al., 2006), there are no national or international studies on its use in the context of nursing practice. This makes the present study pioneering research. The present study was conducted to evaluate the effects of color therapy on fatigue, depression, and quality of life in Parkinson's patients. Due to the lack of directly comparable studies on color therapy for Parkinson's patients, the findings were discussed in comparison with the results of other non‐pharmacological intervention studies in the same patient group.

When the descriptive characteristics of both groups were analyzed, it was found that Parkinson's patients were statistically similar in terms of descriptive characteristics (*p* > 0.05). It is an important finding that the descriptive characteristics are similar, there is no difference in the methods applied between the groups, and the groups are homogeneous.

Mean fatigue scores of the intervention group were significantly decreased. In this context, the result that the control group had increased levels of fatigue due to the presence of disease‐related symptoms and high levels of depression reflects a normal situation. In a study, Parkinson's patients were given Pilates training 3 days a week for 8 weeks, and as a result of this training, Pilates exercises were found to reduce fatigue in Parkinson's patients (Çağlar et al. 2024). In another study, balance and fatigue of Parkinson's patients were found to improve with 60 min of Tai Chi practice 2–3 times a week for 3 to 6 months (Li et al. 2022). The level of fatigue was found to be reduced in Parkinson's patients with different non‐pharmacological methods. The orange color used in color therapy in this study is the light in the wavelength range of 585–620 nanometers in the visible spectrum. This is a color that is noticed the longest by the human eye and reflects its energy (Andrews 2012). In this context, it can be said that color therapy application in the present study reduces fatigue by providing vitality and energy in patients; it is a non‐pharmacological application appropriate for use as a nursing intervention.

The post‐intervention mean depression score of the intervention group was found to be significantly decreased compared to the control group. In a study conducted on Parkinson's patients in the literature, it was found that depression symptoms decreased with at least 10 min of physical activity (walking) for a week (Koç et al. 2021). As a result of the study conducted by Bal in 2019 to examine the effect of color therapy on depression levels, it was concluded that color therapy had a significant positive effect on the depression levels of individuals who underwent color therapy (Bal 2019). Our results were similar to the results of studies using different non‐pharmacological methods. The wavelength of the yellow color applied in color therapy is 565–590 nanometers. It is a color with high visibility from long distances. The yellow color provides motivation and positive energy to individuals due to its high visibility (Andrews 2012). In this context, it can be said that the yellow color we applied in color therapy in the present study decreased depression levels by increasing patients’ self‐confidence and optimism and that nurses can use color therapy as a supportive treatment to decrease the anxiety levels of patients.

The intervention group was found to have a significant improvement in the mean scores of SF36 factors of emotional role difficulty, vitality, mental well‐being, social functioning, pain, and general health sub‐dimensions. Göz et al. (2020) found that 60 min of Pilates performed twice a week for 6 weeks was a relaxing treatment applicable to Parkinson's patients and had an improving effect on the walking speed of individuals. In the same study, with the improvement of the walking speed of the patients, it was easier for them to perform their daily life activities, and their quality of life increased (Göz et al. 2020). In addition to yellow and orange colors with high wavelengths used in color therapy, the color purple, which is considered to be the highest dimension of electromagnetic energy, energizes individuals and promotes new beginnings and a sense of struggle (Andrews 2012). In line with these results, we can say that color therapy significantly improves the quality of life in Parkinson's patients and is a method that can be applied by nurses.

All these results confirm the research hypothesis that “color therapy has an effect on the level of fatigue, depression, and quality of life in Parkinson's patients.” In addition to comparisons with other non‐pharmacological interventions, evidence from studies on bright light therapy (BLT) suggests that light‐based treatments may benefit non‐motor symptoms such as mood, sleep disturbances, and fatigue in patients with Parkinson's disease. A systematic review and meta‐analysis of randomized controlled trials found that BLT significantly improved depressive symptoms and sleep quality in Parkinson's patients (Lin et al. 2021). Evidence from randomized controlled trials also indicates that BLT can improve subjective sleep quality and alter neuroendocrine markers such as cortisol, which may relate to energy levels and daytime functioning (Rutten et al. 2019). These effects are thought to occur through modulation of circadian rhythms via retinal photoreception, which provides signals to the suprachiasmatic nucleus and influences melatonin and cortisol secretion (Paus et al. 2007; Rutten et al. 2016). Although the protocols and outcomes in the literature vary, these findings support a plausible mechanism by which light‐ and color‐based therapies may reduce fatigue and improve quality of life, providing a biological basis for our results and reinforcing the potential of such interventions in Parkinson's care.

### 4.1 Strengths and Limitations

This study has several strengths and limitations that should be considered when interpreting the findings. The absence of similar randomized controlled trials investigating the effects of color therapy on fatigue, depression, and quality of life in patients with Parkinson's disease represents an important strength, enhancing the originality of the study and contributing to the existing literature. In addition, conducting the intervention in a dedicated room under controlled environmental conditions helped standardize the implementation process and minimize potential external influences.

However, several limitations should be acknowledged. First, 3‐month post‐intervention follow‐up data were not collected, as the study design did not include this assessment. Second, the study was conducted in a single center, and the sample was limited to participants from a specific geographical region, which may restrict the generalizability of the findings to the broader Parkinson's disease population. Furthermore, although data analysis was performed by a blinded independent statistician, blinding of participants and the researcher implementing the intervention was not feasible due to the nature of the color therapy application. As a result, the observed improvements in several quality of life dimensions (Role Emotional, Vitality, Mental Health, Social Functioning, Pain, and General Health) may have been influenced by participants’ expectations or suggestion effects. The absence of participant and interventionist blinding may have introduced performance and expectation bias, particularly in subjective outcome measures such as self‐reported fatigue, depression, and quality of life. Therefore, the single‐center design together with the lack of blinding may have limited the external validity of the study results.

Several practical challenges were also encountered during the research process. Mobility limitations and fluctuating cognitive status among Parkinson's patients occasionally affected regular participation in intervention sessions. Additionally, initial hesitancy toward an unconventional intervention such as color therapy required additional motivation and trust‐building efforts by the researcher. These challenges were largely managed through careful planning, individualized support, and effective communication throughout the study process.

## 5 Conclusion

This study showed that color therapy had significant effects on reducing fatigue and depression levels and improving quality of life in Parkinson's patients. After the intervention, a statistically significant decrease in fatigue and depression scores and an increase in some sub‐dimensions of quality of life were found in the intervention group. The findings show that color therapy contributes to the psychosocial well‐being of Parkinson's patients as a complementary approach. In this respect, the study hypothesis was confirmed, and it was concluded that color therapy is an effective method that can be evaluated in clinical practice. Future studies are recommended to include longer‐term follow‐up assessments, such as 3 months post‐intervention, to evaluate the sustainability of color therapy effects in Parkinson's patients. In addition, conducting studies across multiple centers and diverse geographical regions would help enhance the generalizability of findings. Incorporating objective outcome measures and, where feasible, strategies to blind participants and interventionists could reduce performance and expectation bias. Finally, future research could explore methods to support patients with mobility or cognitive limitations to ensure consistent participation in intervention sessions.

### 5.1 Implications for Nursing Practice

Color therapy can be considered a complementary intervention that supports physical, emotional, and spiritual well‐being in nursing care. In line with the findings, nurses can apply color therapy in holistic care processes, especially for Parkinson's patients coping with fatigue, depression, and low quality of life. The fact that color therapy is an easy‐to‐apply, low‐cost, and non‐invasive method makes it possible to integrate it into the daily practices of nurses. However, in order to use this method effectively, it is recommended to increase the knowledge levels of nurses about color therapy and to provide training opportunities and appropriate physical conditions in clinical settings. In addition, including such interventions in the nursing education curriculum will strengthen the understanding of complementary care, support a patient‐centred approach, ultimately enhance the quality of care provided to patients, and promote holistic healing practices.

## Author Contributions

**Kübra Coskun**: conceptualization, investigation, writing – original draft, methodology, data curation, resources. **Gulcan Bahcecioglu Turan**: conceptualization, investigation, writing – original draft, methodology, writing – review and editing, data curation, resources

## Funding

The authors have nothing to report.

## Conflicts of Interest

The authors declare no conflicts of interest.

## Data Availability

The data supporting the findings of this study are available from the corresponding author upon reasonable request.
